# Effects of sewage sludge hydrochar on emissions of the climate-relevant trace gases N_2_O and CO_2_ from loamy sand soil

**DOI:** 10.1016/j.heliyon.2022.e10855

**Published:** 2022-10-08

**Authors:** Arpan Joshi, Marc Breulmann, Elke Schulz, Reiner Ruser

**Affiliations:** a CDRSP – Politécnico de Leiria, Marinha Grande, Portugal; b Institute of Crop Science, Department Fertilization and Soil Matter Dynamics (340i), University Hohenheim, Fruwirthstraße 20, 70599 Stuttgart, Germany; c Helmholtz Centre for Environmental Research—UFZ, Permoserstraße 15, 04318 Leipzig, Germany; d Helmholtz Centre for Environmental Research—UFZ, Theodor-Lieser Straße 4, 06120 Halle, Germany

**Keywords:** Solid waste management, Carbon sequestration, Hydrothermal carbonization, Carbon and nitrogen mineralization, Microbial biomass

## Abstract

This work explores the effects of amending a loamy sand soil with hydrochars having different physicochemcial characteristics. The effects of different hydrochars on emissions of the greenhouse gases nitrous oxide (N_2_O) and carbon dioxide (CO_2_) were investigated together with the relationship between the hydrochar's mineral nitrogen content and the soil microbial biomass. Soil samples were amended with eleven different hydrochars and feedstocks having different carbon and nitrogen contents at application rates of 5 t ha^−1^ and 25 t ha^−1^. Microbial immobilization was the main mineral nitrogen sink in soil following hydrochar application. Moreover, the processing conditions applied during hydrochar production (i.e., the pyrolysis temperature and residence time) had significant effects on N_2_O and CO_2_ emissions: treatment with incubated hydrochars yielded significantly lower N_2_O emissions than treatment with non-carbonized feedstocks, particularly at the highest level of hydrochar application (25 t ha^−1^). Further analysis revealed that increasing the process temperature and residence time during hdyrochar production significantly increased the final product's total organic carbon content but reduced its content of hot water extractable carbon. Hydrochars produced with higher process temperatures and longer residence times therefore yielded lower CO_2_ emissions during a 44-day incubation experiment than raw feedstocks or hydrochars produced under less severe conditions. Hydrochars formed from sewage sludge at high process temperatures and with long residence times are thus promising soil additives for reducing GHG emissions.

## 1 Introduction

The most important drivers of anthropogenic greenhouse effects are carbon dioxide (CO_2_), methane (CH_4_) and nitrous oxide (N_2_O), with gases such as halogens, hydrocarbons, and aerosols also contributing to a degree. This study focuses on the release of two primary greenhouse gases, N_2_O and CO_2_, following the application of hydrochars to soils. Studying the fluxes of these gases is important because of their great contribution to global radiative forcing (Lahn, 2021).

Wastewater treatment plants are major sources of anthropogenic GHG emissions. Efforts to reduce these emissions are complicated by the need to treat and dispose of large quantities of sewage sludge (SS), which has emerged as a significant global challenge in recent decades (Suocheng et al., 2001). Sludge produced during wastewater treatment can be disposed of by incineration or applied to land after composting or digestion. While incineration generally produces higher GHG emissions (Chen and Kuo, 2016), both options can strongly affect environmental quality and sustainable development (Shaddel et al., 2019). The current legal options for SS treatment in Germany include incineration, landfilling, and agronomic reuse as fertilizer in agricultural land, forests, and grasslands. The agronomic reuse of SS may however have several adverse ecological consequences and health impacts because of the huge volumes of SS that are produced and their potentially high contents of toxic heavy metals, various organic chemicals, and pathogens (Shaddel et al., 2019). Consequently, new ways to dispose of SS that are safe, sustainable, and societally beneficial are needed (Schnell et al., 2020). The transformation of SS into hydrochars may be an attractive way of reducing the ecotoxic impacts of SS processing while also reducing the associated GHG emissions (Shaddel et al., 2019).

Hydrochars are formed by the hydrothermal carbonization (HTC) of SS. This process has several possible benefits: among other things, it can remove pathogens, degrade organic contaminants, eliminate or demobilize inorganic pollutants, and improve the overall energy and material balances of sanitation systems (Breulmann et al., 2015). Advances in HTC techniques have expanded the range of feedstocks that can be used in char production, enabling the use of unconventional materials such as wet agricultural residues, human waste, SS, and municipal solid wastes (Ischia and Fiori, 2021). However, relatively dry feedstocks are preferred because the dehydration of wet material is time- and energy-intensive (Dicke et al., 2014). Despite this, the use of organic-based waste products as feedstocks for hydrothermal biomass conversion is attractive because it could reduce waste volumes while also providing new sources of bioenergy (Lee et al., 2019). Accordingly, it has been shown that using HTC to process agricultural and municipal solid waste can be environmentally friendly because it reduces GHG emissions while producing valuable residual materials (Pawlak-Kruczek et al., 2020).

Although hydrochars have numerous benefits when used to amend soils, several studies have reported contradictory results concerning their effects on GHG emissions. Adjuik et al. (2020) found that hydrochars formed from digested SS act as CH_4_ sinks when used as fertilizers and can reduce CO_2_ emissions by up to 34 % relative to control treatments. The application of hydrochars to soil also reportedly has substantial effects on N_2_O emissions but the magnitude of these effects varies widely between studies. For instance, during incubation experiments conducted by Kammann et al. (2012), amendment with hydrochars only reduced N_2_O emissions at the start of the incubation period and significantly increased overall emissions relative to controls—by as much as 302 % and 155% in the cases of hydrochars derived from beet and bark, respectively. These authors also observed a strong increase in CO_2_ emissions following hydrochar amendment. Similarly, in soil column experiments conducted by Chu et al. (2020), treatment with a hydrochar derived from *Chlorella Vulgaris* increased N_2_O emissions 2.2–2.8-fold when compared to a control treatment. However, hydrochar incorporation reduced N_2_O emissions from soil in other cases. For example, Wang et al. (2020) reported that sawdust-derived hydrochar reduced N_2_O emissions from low fertility soils by 26 %–37 % at low application rates. Reduced N_2_O fluxes from soil were also reported following treatment with hydrochars derived from corn silage (Malghani et al., 2013) and for hydrochars derived from wheat straw and digested wheat straw (Dicke et al., 2014). Finally, Zhou et al. (2018) reported that hydrochar treatment reduced the N_2_O emissions of soil columns from a paddy field by 6.1–32.3%.

The inconsistencies in these results suggest that several factors influence the impact of hydrochar amendment on GHG emissions from treated soil. Therefore, this study aimed to determine how soil N_2_O and CO_2_ emissions as well as soil N dynamics and microbial biomass respond to amendment with hydrochars with different physicochemical characteristics derived from different feedstocks.

## 2 Hypothesis

Based on a literature review, an incubation experiment was conducted to test the following three hypotheses:

a. CO_2_ and N_2_O emissions from soils amended with hydrochars depend on the feedstock from which the hydrochar derives, its processing parameters, and its rate of application. CO_2_ and N_2_O emissions decrease as the HTC residence time and process temperature increase.
b. After hydrochar incorporation, the CO_2_ flux increases due to the presence of easily available organic carbon while N_2_O emissions fall compared to controls.
c. Hydrochar amendment increases microbial biomass because it adds labile carbon to the soil.

## 3 Material and methods

### 3.1 Experimental soil

Soil samples were collected from a field at the Berge experimental station (Nauen) of the Institute for Agricultural and Urban Ecological Projects at Humboldt University, Berlin, Germany. The station is located northwest of Berlin (52° 37′ 12″ N, 12° 47′ 42″ E) at an altitude of 45 m above sea level. The long-term average temperature at the site is 8.7 °C and the mean precipitation is 503 mm a^−1^. The initial pH (10^−2^ *M* CaCl_2_) was 6.13 and the soil texture was loamy sand; it consisted of 75% sand, 17% silt, and 8% clay. Soil samples were taken from the untreated control plots of a field experiment at a depth of 0.2 m and mixed thoroughly before being air-dried for 3 days. The dried soil was then homogenized, sieved (mesh size: 2 mm), and stored at room temperature.

### 3.2 Preparation of soil—substrate mixtures

Soil incubation experiments were performed in a thermo-constant climate chamber. The temperature was controlled using data loggers (LogTag, TRIX-8, CIK solutions, Karlsruhe, Germany). The mean, minimum, and maximum temperatures inside the climate chamber during the experimental period were 21.25 °C, 19.3 °C, 23.2 °C. Soil aliquots (300 g) were weighed into 0.5 L glass vessels and compacted to a bulk density of 1.3 Mg m^−3^. The soil was then pre-conditioned for 9 days at a water-filled pore space (WFPS) value of 30 % to restore microbial activity. After pre-conditioning, the soil was post-conditioned at 70 % WFPS with further compaction to a final bulk density of 1.45 Mg m^−3^. Deionized water was added to maintain a WFPS of 70 % during the incubation experiment. The maximum water-holding capacity of the soil was determined according to Ohlinger & Kandeler (1996).

Case et al. (2012) reported that raising the WFPS of soil samples to 78 % greatly increased their N_2_O emissions during the first 72 h following water addition. Moreover, microbial N_2_O sources such as nitrification, nitrifier denitrification, and denitrification are known to be moisture-sensitive, so the soil moisture content is expected to have an appreciable effect on measured N_2_O emissions.

Eight soil additives were examined in the analysis—three feedstocks and five hydrochars. The feedstocks were primary sludge (PS); activated sludge (AS) and straw (ST). The hydrochars were prepared by heating the feedstocks at temperatures between 180 °C and 230 °C for 2–8 h and are referred to as HTC-I, HTC-II, HTC-III, HTC-IV, and HTC-V (Table 1). The feedstock mixture of straw and activated sludge (ST-AS) was only used to produce HTC-V and was not used as a treatment. All additives were homogenized, milled, and stored in closed plastic cups at room temperature until analysis.

**Table 1:** Soil additives used in the incubation experiment and their application rates. PS: primary sludge; AS: activated sludge; ST: straw; HTC: hydrothermal carbonization. The straw and activated sludge (ST-AS) feedstock was only used to produce HTC-V and was not used as a treatment.

Feedstock	Hydrochar	Residence time [h]	Process temperature [°C]	Application rate [t ha^−1^]
PS		-	-	5.0 ​| ​25.0
	HTC-I	2.0	180.0	5.0 ​| ​25.0
	HTC-II	7.0	230.0	5.0 ​| ​25.0
AS		-	-	25.0
	HTC-III	8.0	210.0	25.0
ST		-	-	25.0
	HTC-IV	7.0	230.0	25.0
ST-AS		-	-	-
	HTC-V	7.0	230.0	25.0

### 3.3 Incubation experiment

The incubation experiment included eleven different treatments that differed in terms of the feedstock or hydrochar used to amend the soil and their application rates, as shown in Table 1. The chemical properties of the soil additives are shown in Table 2.

**Table 2:** Chemical properties of the feedstocks and hydrochars.

Feedstock	Hydrochar	TOC (%)	TN (%)	NH_4_–N (g kg^−1^)	HWC (g kg^−1^)	HWN (g kg^−1^)
PS		21.04	2.91	4.35	49.95	10.31
	HTC-I	29.67	3.42	4.43	41.38	11.83
	HTC-II	30.85	2.66	3.11	33.87	9.28
AS		32.59	4.42	7.51	114.26	26.49
	HTC-III	39.29	2.99	3.85	39.85	12.52
ST		46.87	0.25	0.01	31.81	1.35
	HTC-IV	54.99	0.26	0.01	33.52	0.79
ST-AS		-	-	-	-	-
	HTC-V	42.98	3.15	4.42	43.10	13.78

TOC: Total organic carbon; TN: Total nitrogen; HWN: Hot water extractable nitrogen; HWC: Hot water extractable carbon.

Gas chromatography was used to measure the emissions of CO_2_ and N_2_O from samples of a loamy sand soil after mixing with the various additives during the incubation experiment. For this purpose, samples of the pure soil and mixtures of the pure soil with the various additives were incubated in 0.5 L glass vessels. As mentioned previously, the soil samples were pre-conditioned for nine days to restore microbial activity before starting the experiment. The samples were then mixed to homogeneity with either sewage sludge or a hydrochar in proportions of either 0.56 g or 2.88 g per 300 g of dry soil (corresponding to field application rates of 5 t ha^−1^ and 25 t ha^−1^, respectively). The incubation experiment was conducted under aerobic conditions with five replicates per treatment (12 × 5) for 44 days. Soil without additives was used as a control.

### 3.4 Trace gas analysis and calculation of gas flux rates

The flux rates of CO_2_ and N_2_O were measured every 12 h during the incubation period to determine the effects of the additives on greenhouse gas emissions. The headspace above the soil was sampled by flushing the glass vials with nitrogen (N_2_) as an inert gas and then evacuating them at a pressure of around -950 mbar using a rotary vacuum pump (RZ 9, VACCUBRAND, Germany). Before being returned to the incubation system, the vials were restored to normal atmospheric pressure. While sampling the vial's headspace, five additional samples were collected from the inlet gas stream. Trace gas concentrations in the glass vials were measured using a gas chromatograph (GC) equipped with a ^63^Ni electron capture detector for CO_2_ and N_2_O detection (5890 series II, Hewlett Packard) and an autosampler (HS 40, PerkinElmer). The operating mode of the GC system including its back-flushing with water vapour was as described by Loftfield et al. (1997). Flux rates were calculated based on the differences in analyte concentration between the inlet and exhaust air together with the gas flow through the glass vessels' headspace and the air temperature as described by Hantschel et al. (1994) and by Flessa and Beese (1995).

Gas flux rates were calculated from the measured gas concentrations (which were in ppm for CO_2_ and ppb for N_2_O). These concentrations were converted into mass concentrations (i.e., from ppm or ppb into mg CO_2_ m^−3^ or μg N_2_O m^−3^) using the ideal gas law in conjunction with knowledge of the molecular masses of the two gases to obtain final concentrations in units of g m^−3^ for CO_2_ and mg m^−3^ for N_2_O.

The fluxes of trace gases were calculated using the following equation:

(1) Flux _gas_ = K _gas_ × 273/ T × ([Gas] _out_—[Gas] _in_) ×S / A

Here,

Flux _gas_ = flux rate (mg CO_2_–C m^−2^ h^−1^ or μg N_2_O–N m^−2^ h^−1^)
K (N_2_O) = Conversion factor for N_2_O (1.25 μg N_2_O–N μL^−1^)
K (CO_2_) = Conversion factor for CO_2_ (0.536 mg CO_2_–C mL^−1^)
T = Temperature [K]
[Gas] = Gas concentration in the inlet air (ppm for CO_2_ and ppb for N_2_O)
S = Air velocity [L hr^−1^]
A = area of soil in the glass bottles [m^2^]

### 3.5 Soil analysis

The soil moisture content was determined by drying 10 g aliquots of fresh soil for 24 h at 105 °C. The mineral nitrogen content (NH_4_^+^-N and NO_3_^–^N) of the soil was determined for the initial samples (immediately after applying the amendment material) and for samples at the end of the incubation period. To determine mineral N content, 20 g of fresh soil were extracted with 80 mL of a 0.5 M K_2_SO_4_ solution. The concentrations of NO_3_^–^N and NH_4_^+^-N in the filtrates were then determined by flow injection analysis (3 QUAAtro, SEAL Analytical, UK).

Soil microbial biomass was determined at the start and end of the incubation period using the substrate-induced respiration (SIR) method. Substrate saturation and maximum initial respiration responses were determined using an amendment rate of 4.0 mg glucose g^−1^ dry matter. The CO_2_ evolved during the incubation was collected in 0.05 M NaOH (Anderson and Domsch, 1989).

Hot water extractions (HWE) were performed as described by Breulmann et al. (2012). Air-dried soil samples (10 g) were boiled in 50 mL of deionized water (pH = almost 6.0) for 1 h in a reflux condenser using an extraction ratio of 1:5 soil/water (w/v). After cooling to room temperature, 0.1 ml of 1 M MgSO_4_ was added to each suspension, which was then centrifuged for 10 min at 2000 rpm to obtain a clear extract. The extracts were filtered using RC 25 Minisart single-use syringe membrane filter units with a 0.45 μm pore size to eliminate microorganisms (Sartorius AG, Göttingen, Germany). All extracts were analyzed for total C and N concentrations (C-HWE and N-HWE) using an elemental analyzer for aqueous samples (Micro N/C and Multi N/C, Analytik Jena, Germany).

### 3.6 Statistical analysis

All statistical analyses were performed using SAS statistical software (version 9.4). Flux rates of N_2_O and CO_2_ were determined using repeated measures analysis of variance (ANOVA) with a mixed procedure, using the sampling date as a within effect. Gas samples were analyzed using a completely randomized design. The observations were not independent, so a compound symmetry correlation matrix was used to account for correlations among observations. For this purpose, the following linear model was adjusted:

(2) Yi = μ + Ti + βXTi + ei

Here, Yi = observations; μ = general intercept; Ti = effect of treatment; β = effect of slope; X = effect of day; and e = residual error.

Given the model, t-tests were used to compare different treatments and identify significant differences. Where necessary, parameters were transformed to satisfy the requirement for homogeneity of variance and normal distribution. This made it possible to identify significant differences between treatments on the same sampling date and also to identify significant differences between treatments over the test period as a whole. The LSMEANS method was used for mean comparisons. Least significant differences are shown at α = 5%. All figures show untransformed data.

## 4 Results

**Figure 1:**
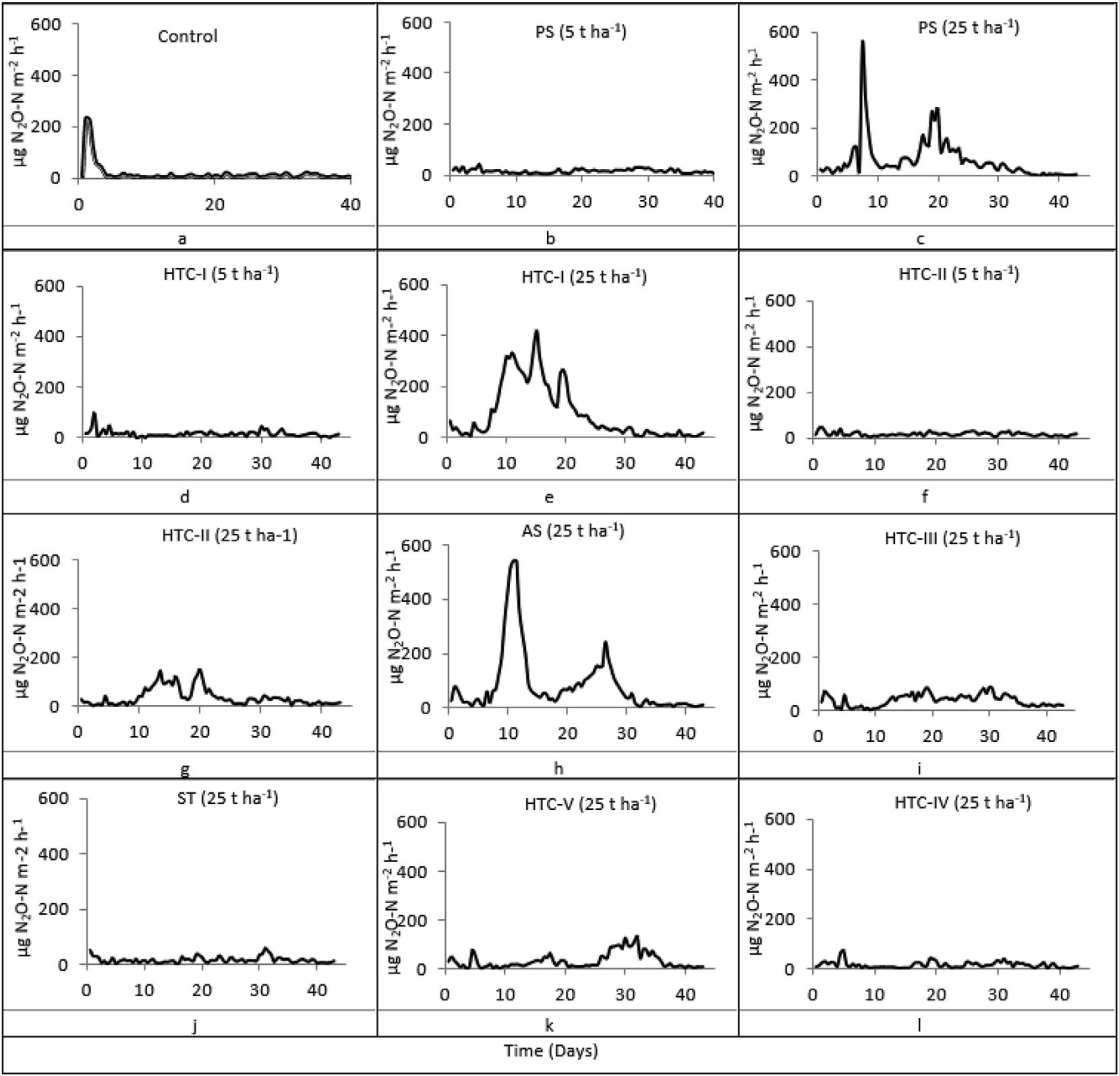
Average N_2_O fluxes (n = 5) for the studied treatments over 44 days of incubation following hydrochar amendment. PS: primary sludge, AS: activated sludge; HTC: hydrothermal carbonization; ST: straw. For more information see Table 1.

The CO_2_ and N_2_O fluxes observed during sampling varied substantially. As expected, the effects of hydrochars on N_2_O and CO_2_ emissions depended on the temperature and the residence time applied during their pyrolysis. Plots of the average daily N_2_O and CO_2_ fluxes (see Figures 1 and 3) for the different treatments show sinusoidal patterns with regular intervals.

### 4.1 Nitrous oxide emission: effects of application rate, feedstock, and process conditions

Figure 1 shows the average N_2_O flux for selected treatments during the 44-day incubation experiment. Flux rates varied widely between the treatments and changed over time. In general, N_2_O emissions increased shortly after applying the additives to the soil. However, in a few cases this increase was weakened as the hydrochar addition progressed.

At an application rate of 25 t ha^−1^, six treatments generated higher N_2_O emissions than the control: PS, AS, HTC-I, HTC-II, HTC-III, and HTC-V. Lower N_2_O emissions than the control were observed following treatment with hydrochar produced from straw (HTC-IV) at an application rate of 25 t ha^−1^ and for PS, HTC-IV, HTC-I, HTC-I, and ST at the lower application rate of 5 t ha^−1^.

**Figure 2:**
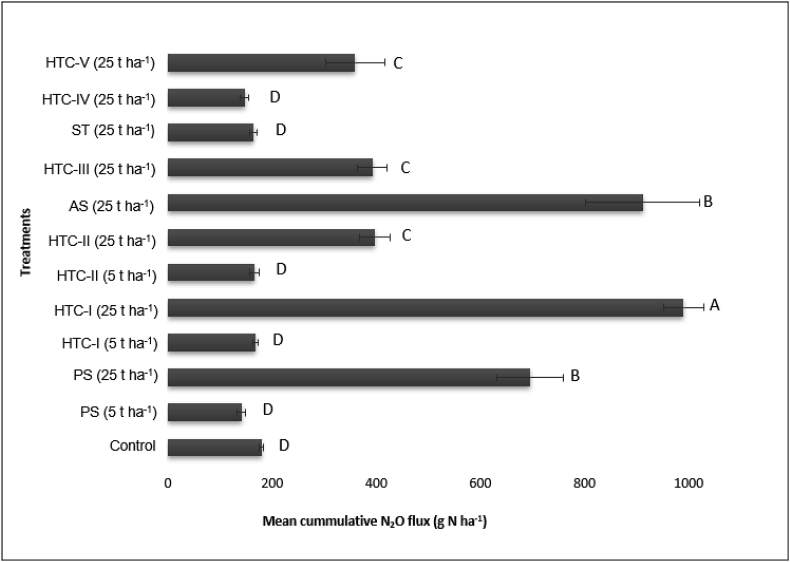
Mean cumulative N_2_O–N emissions (n = 5, ±standard error) over 44 days after different biochar amendments. Statistically significant differences are indicated by different letters (t-test, p < 0.05). PS: primary sludge, AS: activated sludge; HTC: hydrothermal carbonization; ST: straw. For more information see Table 1.

Figure 2 shows the mean cumulative N_2_O emissions for the different treatments after incorporating the various additives. The mean cumulative N_2_O emissions ranged from 141.3 g N ha^−1^ for treatment with primary sludge at 5 t ha^−1^ to 991.2 g N ha^−1^ for treatment with HTC-I at 25 t ha^−1^. Applying hydrothermally treated activated sludge (HTC-III and HTC-V) generated lower N_2_O emissions than direct application of activated sludge when the additives were applied at 25 t ha^−1^. Additionally, the reduction in N_2_O emissions achieved by applying the HTC-IV hydrochar formed by hydrothermal treatment of straw at high temperature with a long residence time (17.6%) was lower than that achieved by applying the straw feedstock itself (8.2%), although the difference was statistically non-significant. Treatment with hydrochar formed by processing sewage sludge at low temperature with a low residence time (HTC-I) led to significantly higher emissions than directly applying primary sludge (PS) when both materials were applied at a rate of 25 t ha^−1^. However, application of hydrochar formed from the same sewage sludge with a higher processing temperature and residence time (HTC-II) led to lower N_2_O emissions than treatment with HTC-I when the application rate was 25 t ha^−1^.

**Figure 3:**
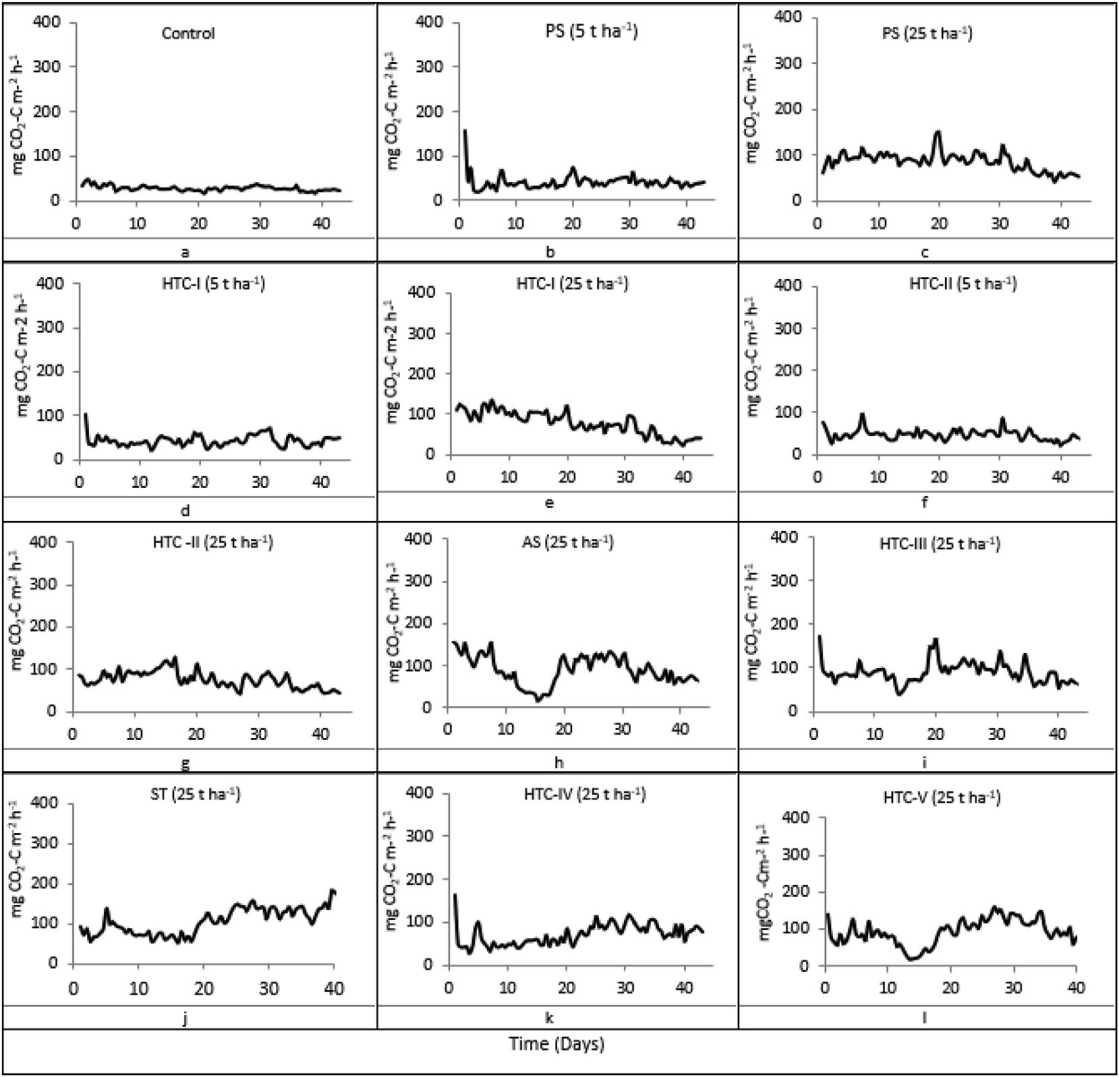
Average daily CO_2_ fluxes (n = 5) after biochar amendment during the 44-day incubation trial. PS: primary sludge, AS: activated sludge; HTC: hydrothermal carbonization; ST: straw. For more information see Table 1.

### 4.2 Carbon dioxide emissions: effects of application rate, feedstock, and process conditions

Figure 3 shows the average daily CO_2_ fluxes from the soil under different treatment conditions. Emissions were generally higher from treated soils than from control samples. Additionally, the CO_2_ flux rates varied after applying hydrochar and declined during the last few weeks of the experiment.

**Figure 4:**
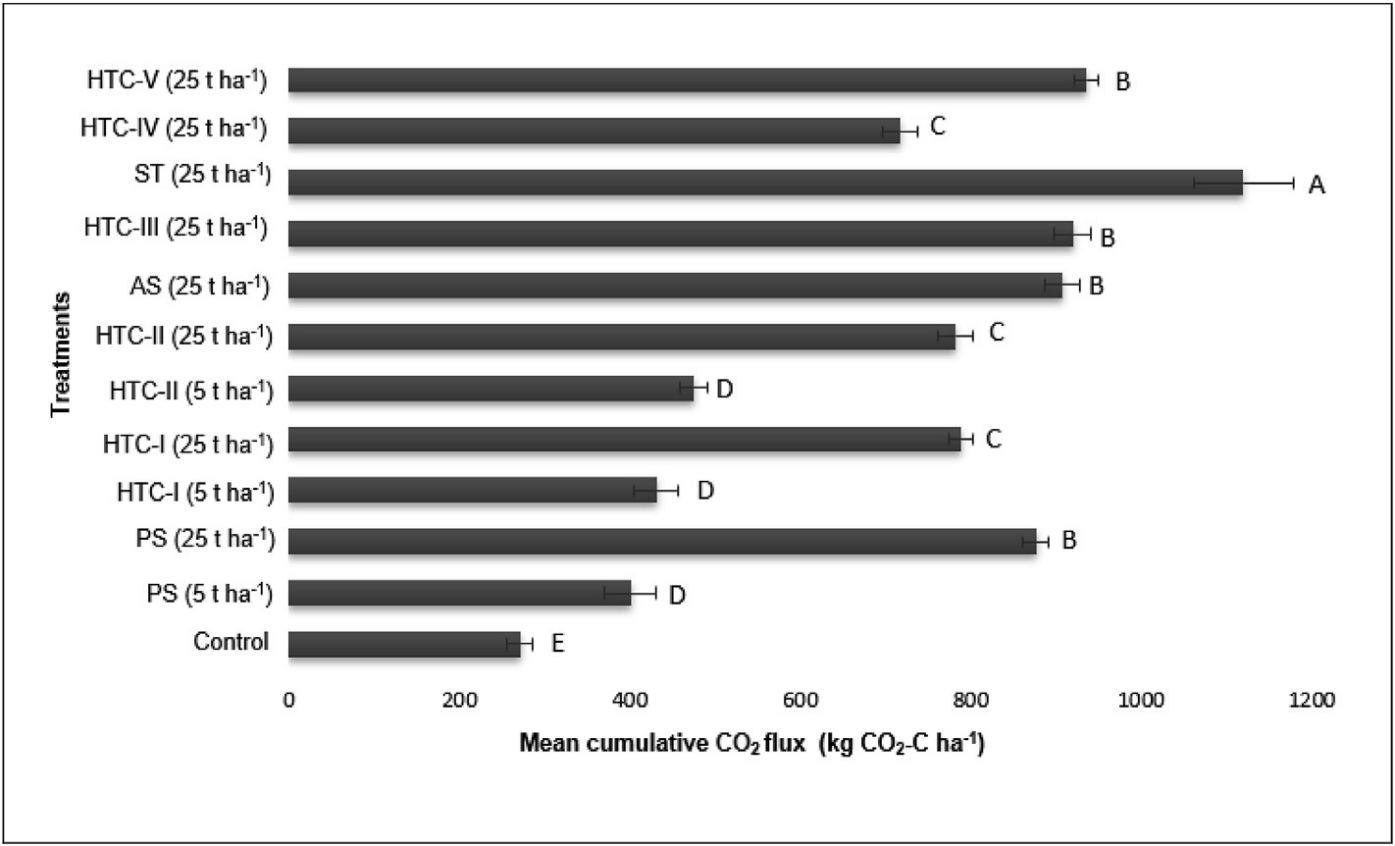
Mean cumulative CO_2_ emissions (n = 5, ±standard error) after hydrochar amendment. Statistically significant differences are indicated by different letters (t-test, p < 0.05). PS: primary sludge, AS: activated sludge; HTC: hydrothermal carbonization; ST: straw. For more information see Table 1.

The mean cumulative CO_2_ emissions for each treatment over the 44-day incubation period are shown in Figure 4. Interestingly, the emissions of samples incubated with HTC-IV were lower than those from samples incubated with feedstock straw. Incubation with hydrochars such as HTC-I generated lower CO_2_ emissions than incubation with primary sludge when the application rate was 25 t ha^−1^. Additionally, incubation with HTC-II yielded lower emissions than HTC-I at this application rate. However, when the application rate was reduced to 5 t ha^−1^ there were no significant differences between the hydrochars and the primary sludge feedstock with respect to CO_2_ emissions.

### 4.3 Mineral nitrogen (N_min_) availability

**Figure 5:**
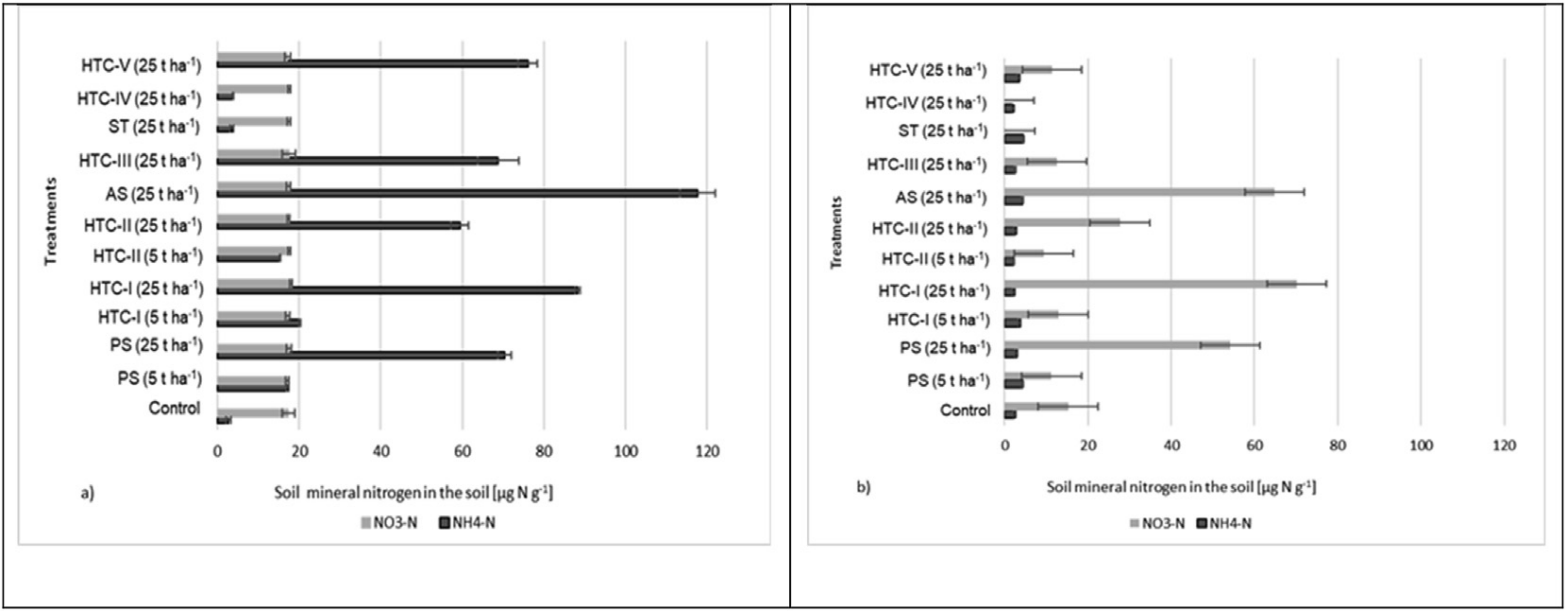
Mean soil NH_4_^+^ and NO_3_^-^ concentrations (n = 3, ± (n = 5, ±standard deviation) for 12 different soil treatments at (a) the start and (b) the end of the incubation experiment. PS: primary sludge, AS: activated sludge; HTC: hydrothermal carbonization; ST: straw. For more information see Table 1.

The mean soil NH_4_^+^ and NO_3_^-^ contents in the 12 treatments (including the control treatment) were evaluated at the beginning and the end of the incubation experiment (Figure 5). At the high application rate (25 t ha^−1^), the highest mean NH_4_^+^ concentrations were measured in samples treated with activated sludge (118 μg N g^−1^) and HTC-I (88 μg N g^−1^). The lowest NH_4_^+^ concentrations were observed in samples treated with ST (3 μg N g^−1^) and HTC-IV (4 μg N g^−1^).

None of the substrates contained nitrate, so the nitrate content at the start of the incubation was almost identical in all treatments. However, samples treated with HTC-I or AS at 25 t ha^−1^ exhibited substantially increased NO_3_^–^N levels (70 μg Ng^−1^ and 65 μg N g^−1^, respectively) at the end of the experiment. Conversely, very low or negligible increases in NO_3_^–^N were observed after adding straw.

### 4.4 Microbial biomass

**Figure 6:**
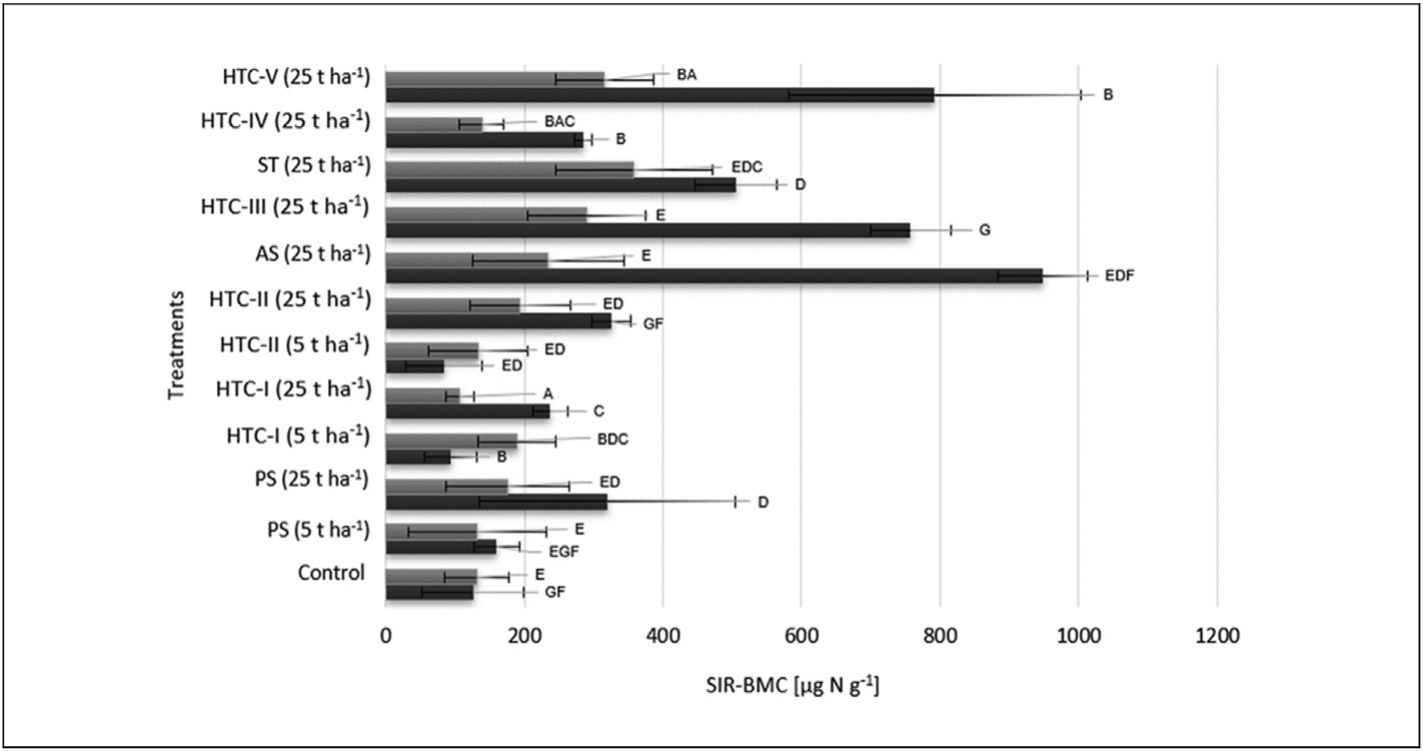
Mean soil microbial biomass determined by the substrate-induced respiration method at the start (t-test, n = 3, ±S.E; black bars) and end (t-test, n = 5, ±S.E; grey bars) of the 44-day incubation experiment. PS: primary sludge, AS: activated sludge; HTC: hydrothermal carbonization; ST: straw. For more information see Table 1.

Figure 6 shows the mean microbial biomass carbon at the beginning of the incubation period (immediately after applying the amendments) and at its end. In all cases, hydrochar amendment significantly increased biomass relative to the control treatment (p < 0.0001). However, the microbial biomass at the end of the experiment was lower than at the start for all amendments other than HTC-I and HTC-II when applied at rates of 5 t ha^−1^. The highest microbial biomass was observed after treatment with AS-25, HTC-IV, HTC-III, and ST at application rates of 25 t ha^−1^. The substrate-induced respiration levels for these treatments were 948, 792, 757, and 505 μg g^−1^ soil, respectively.

## 5 Discussion

### 5.1 Nitrous oxide emissions and N_min_ availability

All additives caused an initial increase in the N_2_O flux, which can be attributed to their content of labile N. At an application rate of 25 t ha^−1^, N_2_O emissions were significantly higher for hydrochars whose production involved shorter residence times and lower processing temperatures (Figure 2), as can be seen by comparing the results for HTC-I and HTC-II. This may be because increasing the process temperature and residence time during HTC reduces the HWN content of the resulting hydrochar and also reduces its mineral N availability due to N immobilization. This decrease in HWN also explains why HTC-III and HTC-IV yielded lower N_2_O emissions than AS and ST, respectively (Table 2). The NH_4_^+^-N concentrations of the soil were also very low in the ST and HTC-IV treatments at application rates of 25 t ha^−1^, which explains the low N_2_O fluxes observed in these cases. The reduction in N_2_O emissions following straw amendment agrees well with the results of Cai et al. (2001).

Amendment with the studied hydrochars and unprocessed feedstocks generally increased the NH_4_^+^ concentration of the soil samples. Figure 5 shows that the ammonium content was highest in soil samples treated with the unprocessed feedstocks AS and PS, and with hydrochars formed from these materials at low temperatures with low residence times. This may explain the high N_2_O emissions observed with these treatments. Amendment with nitrogen-rich sewage sludge or hydrochar derived from such sludge would provide ample mineral nitrogen as a substrate for microbial N_2_O production in soils, leading to high N_2_O emissions.

N_2_O emissions also correlated with the NO_3_^-^ concentrations at the end of the experiment (r = 0.72, n = 12, p < 0.0001). This is consistent with the findings of Kammann et al. (2012), who argued that the high C:N ratio of biochar causes immobilization of mineral N, which serves as a substrate for N_2_O production. Amendment with the tested materials at 5 t ha^−1^ had little effect on NO_3_^-^ concentrations, possibly because the rate of oxygen consumption through C oxidation at this low level of amendment was insufficient to induce anaerobiosis. Conversely, amendment at 25 t ha^−1^ reduced the partial pressure of O_2_ in the soil, making denitrification an important N_2_O source. The hypothesis of increased O_2_ consumption at the higher application rate (25 t ha^−1^) is supported by the high CO_2_ emissions observed under these conditions. Overall, the results obtained clearly show that amendment with sewage sludge increases N_2_O emissions, but this increase in emissions can be alleviated by converting untreated organic material into hydrochar.

### 5.2 Carbon dioxide emissions: effects of application rate, feedstock, and process conditions

Increasing the rate of sewage sludge application increased CO_2_ emissions, probably because it increased the amount of C added to the soil (Carmo et al., 2014). Sewage sludge amendment thus stimulated CO_2_ emissions by increasing the supply of readily available C compounds for microorganisms, leading to increased soil respiration (Focht et al., 1979). Additionally, significant differences between the CO_2_ fluxes for different HTC-based additives were only observed at the high application rate of 25 t ha^−1^.

**Figure 7:**
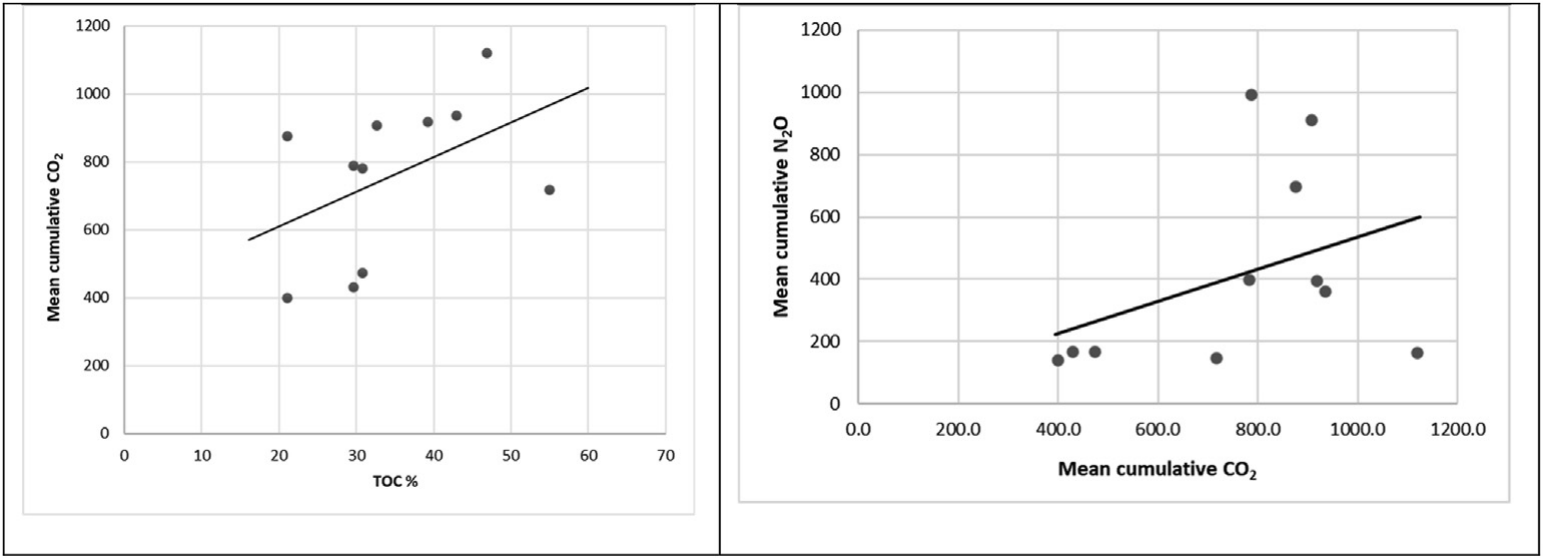
Relationships between total organic carbon (TOC) and mean cumulative CO_2_ emissions (left), and between mean CO_2_ and mean N_2_O emission rates (n = 4).

A moderate positive relationship between N_2_O and CO_2_ emissions was observed after hydrochar amendment (r = 0.43, n = 60, p < 0.05), resulting in the linear trend shown in Figure 7. We also found a positive relationship between CO_2_ emissions and TOC (r = 0.46, n = 4, p < 0.16). The highest TOC values were observed after amendment with straw-based substrates, which may explain the high CO_2_ emissions for these treatments. HTC chars formed at higher temperatures and residence times had lower HWC contents and therefore gave lower CO_2_ emissions than untreated material (Table 2). In addition, increasing the processing temperature and residence time increased the TOC content of the hydrochar relative to that of the raw feedstock. Therefore, applying hydrochar to soil will transiently increase CO_2_ emissionsBreulmann et al., 2018a, Breulmann et al., 2018b showed that this effect can be reversed by washing the hydrochar, which makes it suitable for carbon sequestration. However, this also removes biodegradable compounds and thus reduces the value of hydrochars as soil amendments (Breulmann et al., 2018a, Breulmann et al., 2018b).

### 5.3 Influence of soil additives on microbial biomass

The influence of different soil additives on microbial biomass was determined by the substrate-induced respiration (SIR) method, revealing that the soil microbial biomass was increased to varying degrees following the application of both HTC and sewage sludge. This is consistent with previous reports showing that amendment with HTC increased microbial biomass (Bamminger et al., 2014; Bargmann et al., 2014). The carbon in the amendment material was not just consumed by microorganisms but also incorporated into the microbial biomass, and this was reflected in the substrate-induced respiration values of the treatments. However, in the HTC-I and HTC-II treatments, the incorporation of the added carbon into the microbial biomass was delayed when using an application rate of 5 t ha^−1^, so the microbial biomass at the end of the incubation period was higher than at the start, unlike in most other treatments. This is probably due to a delay in the decay and release of large quantities of carbon incorporated into microbial biomass by microorganisms. It is also possible that the microbial biomass at the end of the incubation period was higher than at the start under these treatments because the applied hydrochars had a lower initial content of labile carbon compounds, causing the soil microorganisms to be C-limited. Regardless of the reason, it seems that these chars were not good food sources for soil microorganisms.

Due to the high carbon availability in straw and activated sludge, treatment with HTC III and HTC V at a rate of 25 t ha^−1^ caused strong increases in microbial biomass together with a clear increase in N-immobilization (see Figure 6). These results thus show that adding sewage sludge and HTC derived from sewage sludge could improve the carbon content of soils and the immobilization of carbon in soil.

## 6 Conclusions

The results obtained showed that the GHG emissions observed after amending soil with hydrochars depends mainly on the process temperature and residence time during the hydrothermal carbonization process rather than the identity of the feedstock material used when producing the hydrochar. CO_2_ and N_2_O emissions were higher for hydrochars processed for 2 h at 180 °C than for those processed for 7 h at 230 °C. This suggests that the hydrochar's content of easily available carbon and nitrogen are important properties with strong effects on short-term emissions of CO_2_ and N_2_O from treated soil. Hydrochar amendment reduced soil mineral nitrogen levels to varying degrees and promoted microbial immobilization, especially when using additives with high C:N ratios such as activated or primary sludge. The risk of causing an undesired increase in CO_2_ and N_2_O emissions by applying hydrochar should therefore be evaluated carefully. The effects of biochar treatment on nitrous oxide emissions clearly depended on both the rate of biochar application and the conditions under which the biochar was processed: in general, higher application rate and more stringent processing conditions (i.e. processing at higher temperatures with longer residence times) gave more favorable results in terms of carbon sequestration and soil amelioration.

We conclude that (i) high application rates are preferable when amending soils with hydrochar derived from activated sewage sludge and straw, and (ii) amending soil with hydrochar derived from sewage sludge is a promising way of reducing GHG emissions.

## Declarations

### Author contribution statement

Reiner Ruser: Conceived and designed the experiments; Analyzed and interpreted the data; Contributed reagents, materials, analysis tools or data.

Marc Breulmaan; Elke Schulz: Conceived and designed the experiments; Contributed reagents, materials, analysis tools or data.

Arpan Joshi: Performed the experiments; Analyzed and interpreted the data; Wrote the paper.

### Funding statement

This research did not receive any specific grant from funding agencies in the public, commercial, or not-for-profit sectors.

### Data availability statement

Data will be made available on request.

### Declaration of interest's statement

The authors declare no conflict of interest.

### Additional information

No additional information is available for this paper.
